# What Psychology Means To Me

**DOI:** 10.4103/0973-1229.27611

**Published:** 2006

**Authors:** Roy Sugarman

**Affiliations:** * *Ph.D. Acting Director of Psychology at Royal Rehabilitation Centre in Sydney, and Conjoint Senior Lecturer in Psychiatry at the University of New South Wales. E-mail: beegone@gmail.com*

**Keywords:** *Psychological Practice*, *Feminism*, *Existential Measning*, *Constructivism*, *Systems Theory*, *Heroism*, *Masculine Identity*

## Abstract

The author takes on the task of describing the interface between emotion and cognition by way of a narrative about psychology, and its meaning to his life. Using time as an overall metaphor, or perhaps a foundation stone underpinning a series of seemingly unconnected events, some insight is given into the author's personal life. The author invokes the works of feminist philosopher and author, Susan Faludi, to portray some aspects of his journey through fantasy, and then the reality of a disparate practice on two continents in psychology and neuropsychology. With particular reference to Faludi's portrayal of men as failed heroes without a role in modern society, the author discovers that all of his work with others has been a work with his own troubled soul, and his failed heroism. Calling on his early role models, and life with and without a sense of purpose, he learns from his clients the value of courage and patience, a spiritual as well as intellectual journey that leads him to become many things to many people in order to heal them, and himself.

## In The Beginning

When I first dreamt up the idea of switching to study psychology, more than 20 years ago, my vision was of a gentle scene: a concerned patient in a chair, a conversation unlike any other taking place against a backdrop of woody desk and book-lined shelf, some brocade was hinted at, wingback chairs.

Years later, surrounded by hundreds of onlookers in an art gallery, I sat next to a naked man on a sun bed while we had a conversation unlike any other.

Let me recount how this came about.

Over the years, I maintained contacts within the artistic community, my wife being one such person. An invitation thus came my way, as part of an installation on the opening night of a new exhibition at a local gallery, to be part of a so-called ′performance art′ scenario, hence finding myself next to a naked man. The artist required of me that I do a full therapy session with him while he lay prone, and nude, on a sun-bed. The metaphor really was that of strong and artificial ′light′ being directed at a man who was ′blind′, conveyed by his wearing blackout goggles, and ′stripped′ of all defence; while a real psychologist sat next to him doing real therapy in terms of a clinical interview and verbal intervention. The whole event was televised. The artist felt the session went well, with certainly some interesting transference-countertransference issues! Then again, the other performance art involved a man pulling the corpse of an antelope around by means of an anal probe tied to the horns, so I am unsure of the merit of the entire thing. However, it was indicative of where psychology, and art, can lead one.

Both of these scenarios, one brocaded, one bizarre, act as bookends to my career, with every other event filling the gaps in between. These gaps, somewhat like holes in the ozone, threaten and cajole, motivating me at times, and at times taking the wind out of my sails.

## Psychology, Time And Personal History

When one tries to fathom what something means or has failed to mean, the natural methodology is to track backwards in time across the history of the personal journey to this point, glancing this way and that to find meaning. Could it be that psychology and the mind are all about time?^1^

Psychology's meaning may thus be about personal history, more than deep hidden value or values, or perhaps more meaningfully, it's a mixture of the two, but unique to each person on either end of the couch.

But let me talk about two other incidents related to the branch out on which I now crawl.

## A Huge Lady On A Bed In South Africa

Instead of being a wise counsellor, seated behind a towering desk in some Manhattan Island dingy apartment at 7 in the evening as in the movies, I sometimes found myself squatting in a basement in the slum land of Johannesburg in South Africa. Next to me was a huge lady on a bed, hysterical and paranoid, while I muttered at her ancestors and cursed mine, mixing some tummy medicine while soothing her, her terrified child looking on.

Upstairs from the tenement, was my office. Working at my desk one night, the young child knocked on my door to say his mother was ‘dying’. This was from a curse her husband’s presumed girlfriend was supposed to have placed on her so she could have access to the aforementioned husband. The assumption of the girlfriend’s existence was based on the husband's recent working hours and overtime, combined with a tummy ache. Given the size of her tummy, the pain was considerable and the assumption fairly reasonable in a very young lady insecure and left alone for long periods with a tiny son. I was the equivalent of a 911 call for him.

Giving a tummy palliative was insufficient, certainly without the melodrama that she required from a healer who would have to deal with the pain of the curse, and the curse itself. A review of her dustbin showed that it was ‘that time of the month’; she was miserable and bleeding; so the administration of a smooth muscle relaxant, and the removal of the ‘overtime’ curse involved mixing the tablet in water, lots of shouting, spitting, the wearing of a buckskin Kaross, and the smooth emulsion of Africa and Europe.^2^

## A Decade Later, In Australia

I will be somewhat less composed and better dressed in Australia, a decade later, when a similar-sized and coloured lady will chase me from her locked ward, after five seconds of assessment.

I sense she is lonely and disenfranchised by the emulsion of Europe and Australia, her children and identity taken away from her. Like a well-travelled suitcase in psychiatry, she is covered with labels; only one side of her functions, and that side is violent. I am afraid of her, so I do not see her again.

I ignore her diagnoses and work with her hatred; I work on her case with her key worker until she is free as she can be as a captive of history and her story. The staff turns on me and seeks to have me removed, to place her in an institution. But she is freed and changes to well and happy and employed. And I see her name in the newspaper a year later, as the new employee of the year.

I wish to celebrate with her, but she does not know me. So I write my joy, confine it to a journal, and leave the evidence base for happiness there.

These two women sit like bells on the bar across the Indian Ocean. Like Atlas, I try to hold up both ends. But I am two different men in two different countries. I shun one, one shuns me, so we are even, if not wise of counsel.

## When Memory Becomes History In Time

My first memories of a counsellor of the wise type turn out to be of a walrus-moustached GP sitting on my chicken-poxed bed; warm hands and cold implements making me wonder what kind of mechanic this was, making home visits to our tiny apartment at the bottom of a mammoth hill in the inner city. We spoke of my plastic helmet and gun bought by a consoling parent. The next morning a real military tank had magically appeared on a plinth at the Returned Soldier's League clubhouse, the only scene visible from my bedroom window. I wondered how the doctor had made that happen. Could it be that these healers worked miracles?^3^

There have been days of magical time: when I have said to a smiling client, “we're done”. Or I have found myself in a sunbeam on a couch, a damaged client cowering in the corner, watching me with pain-dulled eyes as we shifted our ground inch-by-inch, sparring to define the nature of our relationship.

Sometimes in time the setting is not quite as ordinary. Again, my mind goes back to a Boma in the African bush, lions roaring in the close distance, a fire and a bottle of whiskey, and a nurse shedding tears over her HIV-riddled caseload.

The incident goes this way:

As an adviser and coach to a major conservation corporation in Africa, I spent some time with staff in the South African lodges, their numbers dwindling all the time with the highest rates of HIV infection in the world, and mostly very little access to medication. The lodges were intimately associated with the communities in which they existed, and the company tried hard to engage the community on all levels. Suicide amongst those infected was not uncommon. Late at night, with the fire crackling in the Boma, a fenced off dining area, I talked for hours with the nurse charged to manage these endangered people, a memorable encounter with a hamstrung healer. 4

Time travels me across to other continents, and I am mind-transported to a hospital in South Australia, to a room full of medical students, all trying to make sense of the passage of information through the hippocampus. Next door, one of my charges, the 21-year-old King of Israel in fact, waits to find out if a paging unit can set him free from a locked ward after five long years.

This seriously impaired young lad had been kept in locked wards for years against his will, with no remission of his gross psychotic delusions; hence he could not be discharged. His disability however flowed more from his impulsivity and odd behaviour than the psychosis itself, and a small team of us worked to get him out of hospital using an unskilled mentoring system. We also tried, given the nature of his delusions, a kind of ‘mission impossible’ pager that sent him off on buses, shopping, and so on, limiting his impulsivity.

He stayed out of hospital from then on, with the minimal support of a few unskilled workers and a very dedicated community mental health team as well as long-suffering and loving parents. As far as I know, he is still floridly deluded, and always will be. But he is free of us, if not his delusions.

## In Search Of Heroes With My Lantern

How on earth do I make sense of what psychology has meant to me in such diverse scenarios? How do I make sense of what it means to *me*, when me is such a variable in any such equation? How did me become *him*, Dr Walrus, and what miracles did I set out to ‘wrought’?

Susan Faludi, the Pulitzer-prize winning feminist writer perhaps has been the only person brave enough to try to fathom the origin of the male miracle-seeking mechanism.^5^ She found, when examining the narrative over time, that the role model for young boys is not men, but Supermen.

Again, in keeping with her brilliant analysis, I am once again in my bedroom in view of a Sherman Tank. In my bureau are dozens of 1-Penny comic books stuffed with Marvel heroes, not the least of which include *Superman, Batman, Sgt Fury, Spiderman, Fantastic Four*. It does not stop there. For suddenly, I am lying on the grass of a new house, my gaze telescopically raised to the stars where one is moving, slowly, a Russian Hero going where only dogs and chimps have gone before, an evolutionary masterpiece, if I ever saw one. Yuri Gagarin and his ilk will now dominate young boy's fantasies, encased in (a strange word, now common) Sputnik.

Across the world, American boys wait for their heroes to take off from Canaveral, to inspire them to attempt heroism in their own fishbowls. While Alan Shepard is the first astronaut, John Glenn becomes the first American to follow in Gagarin's shadow, and so American would-be heroes find another silvery dot to follow into their dreams.

For Faludi, far from freedom, fame and fortune, little men in the making fantasise of such glory, but not of the fires, the floods and the prosaic famine that will dominate their adult world.

I grew up with a narrow sense of the world and its rules, in a family likewise fettered by apartheid and money problems, and a keen sense of who I was at any given moment, and little understanding of who I could not be. As predicated by Faludi, I rapidly learned to hide inside my head where my putative heroism was safe at all times from turgid reality, and if not, I could make it so. As my life floundered from time to time on the rocks of uncertainty generated by reality and its confrontations, I wondered if only chameleons survived.

Could it be that in order to achieve such heights as the Walrus personified, we need to be nothing to ourselves in psychology, but everything to others?

Instead of being something to me, when I began to look to psychology, I learned to be everything to countless others. Like the comic character superheroes, I needed to stretch endlessly, become invisible, catch on fire, and become a tank-like armoured being. Survival as a hero in psychology is more than Woody Allen's take on being Leonard Zelig, a man for all seasons, everyman's everyman: it becomes a transport of imagination to sustain oneself up sunbeams, down to dungeons, from window to window in a classroom, out of the window, in a hurry, in a hospital with blood on the carpet.^6^

## Slipping The Bonds Of Family Without Fanfare

My parents′ view of what heroism meant was challengingly disparate from mine. So much so that the essence of early heroism turns out to be the courage to divert from the parental pathway to one's own without seeming to be a rebel without a clue.

Psychology slipped seamlessly into my professional instincts at age sixteen, but collided with a maternal fear of female professions. This was not a job for males, esoteric as it was; it could only be for dreamers and fools, and those who did not read Faludi.

So taking the path of parental wisdom, drifting into other trades and professions for a decade, finding all of them hopelessly entangling and impoverished, with a dread of failure looming large, independence came with a young girl who worked with me. She hovered over a blank A4 pad trying to finish a final year essay where she had to write what psychology meant to her. I asked her if I could write it for her, and did some of it to show her I could. Her response led me back to my instincts, complete with desk, bookshelves, wingbacks and brocaded curtains. I could do this, but perhaps I could sally forward without the moustache?

## Psychology, The Chameleon, Listening And Storytelling

Psychology came to mean I could do this. Psychology meant becoming more than a Zelig, more than a shameless parody of others. Psychology meant learning how to become a true chameleon; seamlessly blending in with damaged others, independent of the brocaded accoutrements that warmed Freud's room.

I discovered that becoming a hero to myself was impossible, but that urging others onward and upward was not impossible. Psychology meant learning how to listen on several levels, a unique and invaluable style of listening, and rather than pinning down the diagnosis, it was a method of listening without attribution.

Psychology meant how to get someone with no skills to become an expert and shameless storyteller of a unique script, entirely and copiously his own, stretched across the time of his life.

## A Drunken Psychiatrist, Sartre And Camus

And that reminds me of another incident.

I am suddenly drunk with a companion who is the elderly and first Psychiatrist in my hometown. In our red-wine driven philosophy, and drunkenly drawing on Sartre and Camus, we two push this rock of human pathology up a hill, and when it tries to crush us this night, we agree that depression is about time; that all things mental and mindful are about time and its passage, not about reality but about constructions. Let me explain before you decide it was some drunken nonsense.

We got drunk at a party, and in keeping with what I mentioned before about the executive functions in man, we spoke a lot about time. The conversation drifted to existentialism. The Myth of Sisyphus, quoted by both Sartre and Camus, included for us a putative understanding by Sisyphus (surely!) of events across time, or he would have let go the rock, we presumed. He understood (surely!) that giving up on pushing would result in his being crushed, but also that he could never succeed in shifting the rock upwards and onwards. The resultant ambivalence would result in depression (surely?). Emotion and cognition combine to make neurons produce mind, and mind produce all things mental, such as elation and depression, across time, bound by fantasies of success, perhaps hubris, but it is all about how we construct reality. We wondered what might be passing through Sisyphus's mind as he pushed on his rock.?

## Psychology And The Construction Of Time

Psychology meant the capacity to bend reality without changing the facts, the skill of framing a picture one has not painted, and holding that frame up with no painting inside. I learned not to sit across a desk in a plush-facaded room, but to create a Disneyland where people could play at being uniquely themselves. I heard not voices, but sensed the bindings of time that tied the social knots that created their cognitions. I learned the joy of patience as I waited for their constructions to make sense, for the Shangri-La to come out of the mists of confusion in their first narrative. 7

I learned the real estate agent's skill of immediate summation, of showing them the house that they did not see, on land they did not own, of the potential for renovation of damaged goods. Of their capacity to strut out and win the war the way heroes do when confronted with their own monstrous constructions.

More than anything else, I saw magical comeback games from the abyss, saw houses built without tools or land, saw death and destruction of bad ideas and the love that this brings.

Pages passed my eyes, an endless pack of parading cards in a Stevenson version of a Carrollean dream.^8^ I faced down horrifying evil and devastating loss as a florist would, arranging the stems in a vase filled with blood.

When the esoteric became too vague, and as in the words of Paul Dell, 9 I fell through the ceiling of ontology, I became a Neuropsychologist over-fascinated with systems and feedback loops and minute chemical data of mind that stupefied my capacity to absorb it all. Now I found stunning tales of heroism in Luria's broken worlds, in Goldstein's abstract attitudes, in Prigatano's shining hairdo and Lezak's bibles.^10^

Like Prometheus, I searched out and found hubris and men of integrity, heroes and cowards, my lantern the narrative over time.^11^ Who were they, what happened to them, what changes did they find, what changes did I find, and what to do, what to do, always in time, past's past present and future, the unstoppable passage of time?^12^

Watching them I could track to the final nanosecond of their happiness, that minute moment in time when their heads began their final and hopeless movement of acceleration to unhappiness.

## Maryna And Marie

And again I remember. I am in a room with Maryna and Marie, daughter and mother. I have gone from being my mother's shining light to being her burden. I want my life back. In the right side of her skull, a dent the size of a melon. I want my future back, wrestled from my past. I say okay, we can do this; they leave my room filled with sunbeam and rainbow, their eyes full of tears.

But, first things first. Who are they, Maryna and Marie?

Maryna was a young and brilliant woman with her whole life ahead of her, poised on an astonishing career after a lifetime spent studying the law. Devastated by a traumatic brain injury and epilepsy, she was left with nothing, and began to mutilate herself. Her mother and Maryna came to see me to treat her ‘mental illness′; relieved when I told them this was brain injury, not madness. Some three years later, without much of her right frontal and temporal lobes, she graduated university with distinction, worked as a lecturer, married, and has a doting husband and a lovely baby. Although her tragedy was immense, and I abandoned her to immigrate to Australia, she and her mother persevered and they drove her from darkness into light. Even if she never achieved at the level she had before, her achievements were astounding in the face of the devastating injury. The difference I made to her was explained in an article we wrote together: “The difference was that you believed in me when I needed it the most”.

## Psychology, Heroes, Cowards And Therapy Of The Self

Curiously, when such devastation is presented to me initially, and the client is at the bottom of the pit, I soar inside, psychology giving me the skills, telling me what to do when others, and perhaps even I, can do nothing.

Why am I so happy to see their tragedy come to me? I realize that I cannot be a hero, but can I be the maker of heroes? If I cannot do, then I can teach, and in teaching, live vicariously. I discover a world of heroes in the making, waiting, for in order to become a hero, one must find tragedy, and I wait for it to stun them into me. I have become a vulture of misfortune, trafficking in human misery, and it fills me with joy. I am ashamed of my cowardice in the face of their strength.

In the midst of her therapy, two years later, I left Maryna, but she carried on without me, until she had her life back. What I started I did not finish, leaving the field before the battle was won, a true coward, leaving her a true and startling hero.

Psychology means to me what I mean to me, all therapy is therapy of the self, all treatment of myself, and all words are for me. Psychotherapy became a conversation about time and its passage, and the pathology of its stickiness or fluidity.^13^

Time and travel, pages of it, documenting each slip and skip as we bind events across time and across the timing, and pacing of spacing the words as they come out and are immediately taken up by transport systems, never to come again. And then they come again, and I take my every breath with them, between them and me. Psychology is the homeostatic concern of the universe and its priesthood, the tower of psychobabble its personal hell. The cowardice of facing true heroism.^14^

## Concluding Remarks: In Trying To Remember With Meaning, We Change What We Observe

As I write this, the sunbeam enters my room again, to tell me it is 2h 30 and time to re-elect my past patients and their tangled webs to my consciousness mind. Six thousand faces swirl up behind my eyes and stick and go in their own fashion, unique to them if I let them be. I can, however, stab out and fix one to the background, press their button and watch them live again, each in their own capsule of reality. But in observing them, they change, and are no longer what they were before I fixated them. So I hurriedly unpin their hopes and they charge off like the White Rabbit back into my travelling storage where I keep them safe.

To look upon psychology is to change it, to pin it into each cross-sectional instant where it never resides, not for a second. Psychology is a life's journey of constant and wavering homeostatic listening.

When I hear of the stations around the world that sit and listen constantly for signs of alien life against the background noise left over from creation, I feel this is right, that this is me, listening to psychology, and what it means to me.

## Questions That This Paper Raises

1. What vital elements of a conversation between two people qualify the interaction as psychotherapeutic?
2. Do non-western cultures all agree, as a universal, that there are speaking cures for illness?
3. How do humans change, what are the obstacles?
4. What is the best method across all cultures for change to be sustained?
5. Is psychotherapy and its methodology entrenched in the process of human evolution?

## About the Author

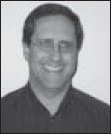



**Dr Roy Sugarman** is a Clinical Psychologist and Clinical Neuropsychologist trained in South Africa and now living in Sydney, Australia. In 2000 he was appointed Senior Neuropsychologist at Royal Adelaide Hospital, and a tutor in Neuroscience, as well as Clinical Lecturer in Psychiatry at Adelaide University. Transferring to Sydney in 2003, he was appointed Principal Psychologist in South Western Sydney, and Clinical Director of the Anxiety and Depression clinical therapies programme at Liverpool Hospital. He is currently Acting Director of Psychology at Royal Rehabilitation Centre in Sydney, and Conjoint Senior Lecturer in Psychiatry at the University of New South Wales. He maintains a private clinical practice and lecturing practice as MLR Consulting Psychology, and Level-7-Psychology in Bondi Junction in Sydney and Adelaide.

## Footnotes

1. In evolutionary terms, the executive functions evolved to include the capacity to bind events across time, allowing us to simulate the world, and choose behaviours now that influence later outcomes favourable to us, rather then choosing the prepotent response now in ignorance of what might be the best temporally future outcome, good or bad. So in this way, I wonder if mind emanates from brain around a scaffold of events in time, and we come to know ourselves as a continuous being because of this capacity?

2. The next day an appreciative husband thanked me for my intervention, when he came off his overtime shift. I suggested he be more attentive: he asked if I was qualified in Witch-doctoring. The aftermath of the story was that my loud replaying of the scene for my family's edification resulted in my sister-in-law's new boyfriend hurriedly leaving our company without eating his dinner, never to be seen again. I think it was too much for his stomach.

3. I discovered later on that I had slept through the arrival of a huge tank transporter that carried on its trailer a fully restored WW II tank. This was placed on a plinth, facing North Africa, its gun aimed up to the sky. With my helmet and gun, bought as presents from my father to make me feel better about being sick, and the tank across the road now appearing magically, I embarked on the first of countless fantasies about being a soldier-hero.

4. A short time before, one of the rangers, facing his own doom at the hands of Africa's greatest scourge, shot himself with his rifle in full view of the dining hall, for which trauma I had to supply counselling and support to the staff. This particular nurse had to deal with that event again, I am sure.

5. Susan Faludi interviewed men across America, including Sylvester Stallone, in order to write, from a Feminist point of view, of how many now felt insecure in their masculine roles in modern society. See Faludi S. (2000), *Stiffed: the betrayal of modern man,* Vintage Books, London.

6. In 1983, Woody Allen produced and acted in a movie about a man, Leonard Selig, a film produced as a narrated fantasy about a man who can look and act like whoever he's near, a human chameleon. He meets various famous people and takes on their identity. A psychiatrist, Dr. Eudora Fletcher, notes how he takes on the persona of real famous people such as Babe Ruth and tries to heal him.

7. Michael White, the great Adelaide narrative therapist helps guide the narrative of patients as they tell their stories, moulding it and collaborating with them until the confused and often meaningless narrative coalesces into the clarity with which the successfully treated patient hopefully departs. Perhaps this is sometimes a pure construction of happiness, a utopia such as Shangri-La, but we live in the world of perceptions, and as therapists we have to be patient as we facilitate the patient constructing their own private utopia.

8. Robert Louis Stevenson wrote *Robinson Crusoe*, about a man abandoned to himself on an island, searching for a companion, an alter ego of himself, his Man Friday, really his own creation but not quite like himself. Lewis Carroll of course wrote *Alice in Wonderland*, the female counterpart, much more coated in fantasy than Crusoe's grim reality, the queen's soldiers really just a pack of cards. What if they collaborated on a project, and Stevenson wrote a dream like Carroll's?

9. Paul Dell was one of the great and early exponents of second order cybernetics in human systems theory, and addressed issues of epistemology and ontology in confronting the clinical views of psychiatry in the ‘80′s. See for instance: **Dell P. (1982)**, In search of truth: on the way to clinical epistemology, *Family Process,* **21**, p407-414.

10. George Prigatano is one of the great figures in the rehabilitation of traumatic brain injury in the USA, with groundbreaking work in self-awareness of deficit. Prigatano is as much known for his work as for his dapper appearance! Alexandra Luria, together with the great Lev Vygotsky, was one of the founding fathers of clinical neuropsychology in Russia and the world. One of Luria's great works tells of a soldier with a ‘shattered world’ who has to relearn all of his history and past world, and recreate himself (see *The man with the shattered world,* Harvard University Press, Cambridge Mass, 1987, first published in 1971). Kurt Goldstein developed similarly on the other side of the world to Luria, and coined the phrase ‘the loss of abstract attitude’ in describing in 1926 the effect of damage to the executive systems of the brain. Muriel Lezak, in serial editions with Oxford University Press, produced in Oregon USA, what is now known widely as the Bible of clinical neuropsychological assessment.

11. Prometheus, of Greek Mythology fame, stole fire from Zeus and gave it to mankind to warm and illuminate them. He was terribly punished for this theft, and Zeus then created Pandora to undo the good work that Prometheus had done. Prometheus felt that all of this was an indicator that the gods had lost touch with mankind, and with their own hearts, and were thus unfit to rule. Prometheus searched for an honest man to break him from his shackles. Here, I refer to the illuminating effect the client's narrative creates over time, shining the light on myself, and the client, both.

12. By “past's past” here I am reflecting on a principle of how memory is laid down. It seems, from studies of those who have both hippocampii removed from their temporal lobes that memory is laid down in the long term over perhaps 5-11 years or so. So there is the past, up to about 10-11 years ago, and then there is the long term past, perhaps from 11 years ago to even beyond ‘*then*’. “*Past's past*” then refers to information so robust we can never lose it, and setting this up against the more ephemeral immediate past, present and future.

13. In keeping with the above, when one is challenged, one turns to the literature, to one's fathers and peers in psychology. Working with the client means working with yourself, inevitably, they grow as and when and if you do, passing on what you learn, challenged by them to learn it. When the client is stuck, when the client flows, in terms of their narrative, the psychologist is there, either patient, or running with them, or pensive in the hours between sessions, anticipating, waiting, learning and growing, for the next challenge.

14. In a way, therapy is designed to perturb, challenge, and to console, following the tenets of dynamic homeostasis, laid down in second order cybernetic principles. If one is not religious, this is the cosmology of change, real and meaningful, not based on the divisions between psychiatry and psychology, or psychological and neuronal, mind or mentality, hence the term tower of psychobabble, or babel more correctly in another form of analogy. In this way we use psychology's weapons to remain calm, while client's cry or struggle; and this is true heroism, to not cry or collapse in the face of life's huge potential for tragic outcomes, but to give your true nature up to meet their heroic struggles in your consulting room. Our clients are so brave.

